# Vitamin D Counteracts Lipid Accumulation, Augments Free Fatty Acid-Induced ABCA1 and CPT-1A Expression While Reducing CD36 and C/EBPβ Protein Levels in Monocyte-Derived Macrophages

**DOI:** 10.3390/biomedicines10040775

**Published:** 2022-03-26

**Authors:** Mirko Marino, Samuele Venturi, Cristian Del Bo’, Peter Møller, Patrizia Riso, Marisa Porrini

**Affiliations:** 1 Department of Food, Environmental and Nutritional Sciences (DeFENS), Division of Human Nutrition, Università degli Studi di Milano, 20133 Milan, Italy; mirko.marino@unimi.it (M.M.); samuele.venturi@unimi.it (S.V.); patrizia.riso@unimi.it (P.R.); marisa.porrini@unimi.it (M.P.); 2 Department of Public Health, Section of Environmental Health, University of Copenhagen, 1014 Copenhagen, Denmark; pemo@sund.ku.dk

**Keywords:** calcitriol, fatty acids, foam cells, C/EBPβ, PPAR-γ1, ABCA1, CPT-1A

## Abstract

The biologically active form of vitamin D, calcitriol (VD3), has received great attention for its extraskeletal effects, such as a protective role on the cardiovascular system. The aim of the present work is to test the capacity of VD3 to affect lipid metabolism and fatty acid accumulation in an in vitro model of monocyte (THP-1)-derived macrophages. Cells were treated for 24 h with oleic/palmitic acid (500 μM, 2:1 ratio) and different VD3 concentrations (0.1, 1, 10, 50 and 100 nM). Lipid accumulation was quantified spectrophotometrically (excitation: 544 nm, emission: 590 nm). C/EBPβ, PPAR-γ1, CD36, CPT-1A, and ABCA1 protein levels were assessed by ELISA kits at different time-points (1, 2, 4, 8, and 24 h). VD3 at 50 and 100 nM significantly reduced fatty acids accumulation in macrophages by 27% and 32%, respectively. In addition, tested at 50 nM, VD3 decreased CD36, PPAR-γ1, and C/EBPβ, while it increased ABCA1 and CPT-1A protein levels in free fatty acid-exposed cells. In conclusion, VD3 reduced fatty acid accumulation in THP-1-derived macrophages exposed to lipid excess. The anti-atherogenic effect of VD3 could be ascribable to the regulation of proteins involved in lipid transport and clearance.

## 1. Introduction

Vitamin D (VD) is a fat-soluble vitamin whose chemical structure derives from cyclopentanoperhydrophenanthrene, the same structural root of steroid hormones [1]. The human body produces VD in the skin by converting 7-dehydrocholesterol to pre-VD upon exposure to ultraviolet-B (UVB) sunlight (290–320 nm wavelength), which undergoes non-enzymatic isomerization to produce VD [1,2,3]. VD is then converted in the liver by 1 or 4 cytochrome P50 microsomal enzymes to the most stable form of VD (i.e., 25(OH)D3) in the circulation, which is finally converted into the active form, calcitriol (1,25(OH)2D3) by the kidneys and other tissues such as colon, lung, breast, prostate and immune cells. [4,5,6,7]. VD can be obtained from vegetal dietary sources in the form of ergocalciferol (e.g., mushrooms, yeasts, and fortified foods) but, above all, from animal foods in the form of cholecalciferol (e.g., fish, egg yolk, meat, offal, and fortified foods) [8,9]. Dietary sources of VD and vitamin tablet supplements are particularly important for populations who live in areas with little sunlight (e.g., Nordic countries) and groups who have higher requirements such as children, pregnant women, the elderly, and patients with certain diseases such as osteoporosis and chronic kidney disease. The VD status in humans is based on the serum concentration of 25(OH)D3 as it reflects intake and is more stable than the active form (1,25(OH)2D3). An interesting cross-sectional study of subjects from the general population in Denmark demonstrated a 20% prevalence of subjects with insufficient serum VD levels in the winter months [10]. The lower levels of VD in the winter months are attributed to low levels of incoming sunlight and clothing (due to cold weather), and VD levels are not completely replenished by the dietary intake of, especially, seafood.

An adequate status of VD is crucial for the normal activity of many organs and tissues. In this regard, the Endocrine Society Clinical Practice Guideline has set the optimal VD serum level at over 30 ng/mL (75 nmol/L). Serum levels below 20 ng/mL (50 nmol/L) reflect a deficiency that could lead to rickets in childhood or osteomalacia and a higher risk of osteoporosis in adults [7,11,12]. In addition, a moderate and severe deficiency appears to increase the risk for atherosclerosis, metabolic syndrome, CVDs [13,14,15,16,17,18], and mortality for CVDs [19,20]. VD affects the redox balance, platelet aggregation, and regulation of the innate and adaptive immune system by preventing infections and autoimmune disorders. [21,22,23,24,25,26,27,28,29,30]. Furtherly, VD has been shown to lower total cholesterol, triglycerides, and LDL-cholesterol and to increase HDL-cholesterol in plasma, thus playing an important role in lipid metabolism and atherosclerosis [28].

It is widely recognized that the hallmark of atherosclerosis is an accumulation of lipids in macrophages and foam cells, which are located in a pro-inflammatory microenvironment of the intima in arteries [31]. The oxidation and modification of the lipid structure represent relevant changes in terms of CVD risk [32]. In addition, the profile of sphingolipids and phosphatidylcholines contained in LDL impacts the susceptibility of their aggregation, which confers a higher risk of future CVD events [33]. Oxidation of LDL (ox-LDL) leads to the activation of the adaptive immune response, mediated by B- and T-cells that enhance the progression of atherosclerotic plaques [34]. In addition, ox-LDL has a higher affinity to plaque macrophage scavenger receptors, resulting in an increased intracellular lipid accumulation [35]. Moreover, the modification of LDL induces the inflammatory process through NLRP3 in macrophages and the consequent detrimental effect through apoptosis and necrotic core development [36]. Additionally, alterations of lipoproteins are responsible for calcium deposits in the arterial intima, which determine a negative effect on hemodynamic pressure and alter viscosity and elasticity of blood vessels, contributing to plaque progression [37].

**Figure 1:**
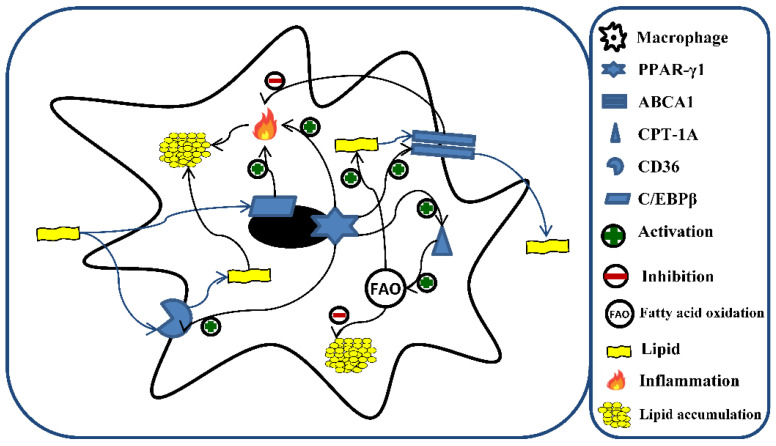
Role of C/EBPβ, PPAR-γ1, CD36, CPT-1A, and ABCA1 in lipid metabolism and fatty acid oxidation (FAO) in macrophages. The excess of lipids is responsible for the induction of different genes involved in the metabolism of fatty acids and the inflammatory process. A central role is represented by PPAR-γ1, which orchestrates the storage and mobilization of lipids through the activation of CD36, CPT-1A, and ABCA1. Additionally, lipids lead to the activation of CD36 and C/EBPβ, which promote intracellular lipid accumulation through the enhancement of lipid uptake and inflammatory response, respectively. Moreover, the activation of CPT-1A, and ABCA1 by the excess of lipids counteract the accumulation of the latter through the stimulation of lipid efflux and increment of fatty acid oxidation, respectively. C/EBPβ (CCAAT enhancer-binding protein beta); PPAR- γ (peroxisome proliferator-activated receptor gamma); CD36 (cluster of differentiation 36); CPT-1A (carnitine palmitoyltransferase 1A); ABCA1 (ATP-binding cassette transporter 1).

Numerous are the potential mechanisms implicated in lipid accumulation. Among them, peroxisome proliferator-activated receptor gamma (PPAR-γ) represents the main regulator of fatty acids storage and mobilization of lipids. However, PPAR-γ interacts with other proteins involved in lipid metabolism (Figure 1). For example, the cluster of differentiation 36 (CD36) [38], devoted to the uptake of lipids, ox-LDL, and their accumulation [39]; CCAAT enhancer-binding protein beta (C/EBPβ), which regulates the progression of foam cell formation and the inflammatory response induced by lipid accumulation [40]; carnitine palmitoyltransferase 1A (CPT-1A) [41], involved in FA oxidation (FAO) [42]; ATP-binding cassette transporter 1 (ABCA1) [43], devoted to maintaining cellular cholesterol homeostasis and preventing foam cell formation through the removal of cellular lipids [44,45]. In addition, ABCA1 has been recognized to exert an anti-inflammatory activity [46].

However, the mechanism through which VD is able to impede the development or progression of atherosclerosis is unknown. We hypothesized that VD could play an important role by reducing lipid accumulation through the regulation of the genes involved in lipid metabolism. Based on this hypothesis, the aim of the present study is to evaluate the effect of the active form of VD on the lipid accumulation in monocyte-derived macrophages THP-1, as a cellular model of atherogenesis, by evaluating the main key proteins involved in the uptake, accumulation, metabolism, and efflux of lipids such as PPAR-γ1, CD36, C/EBPβ, CPT-1A, and ABCA1.

## 2. Materials and Methods

### 2.1. Chemicals and Reagents

Fetal bovine serum (FBS; CAS No. F4135), Hanks’ balanced salt solution (CAS No. H6648), phorbol-12-myristate-13-acetate (PMA; ≥99% TLC; CAS No. P8139), bovine serum albumin (BSA; CAS No. A9418), palmitic acid (≥99% GC; CAS No. P0500), oleic acid (≥99% GC; CAS No. O1008), standard of VD3 (calcitriol; ≥99% HPLC; CAS No. D1530-10UG), Trypan Blue (CAS No. T8154), hydrochloric acid (37%; CAS No. 320331), methanol (≥99.9% HPLC; CAS No. 34860), ethanol (EtOH; ≥99.8% HPLC; CAS No. 51976), pluronic F127 (CAS No. P2443) and Nile Red (≥97%; HPLC CAS No. 19123) were obtained from Merck (Darmstadt, Germany). RPMI-1640 medium (CAS No. 21875091), sodium pyruvate (CAS No. 11360070), 4-(2-hydroxyethyl)-1-piperazineethanesulfonic acid (HEPES; CAS No. 15630106), gentamicin (CAS No. 15710064), and trypsin–EDTA (CAS No. 25200056) were purchased from Life Technologies (Monza Brianza, Italy). Enzyme-linked immunosorbent assay (ELISA) kits related to PPAR- γ, C/EBP-β, CD36, CPT-1A, and ABCA1 were purchased from MyBioSource (San Diego, CA, USA), while ultrapure water was from a Milli-Q apparatus (Millipore, Milford, MA, USA).

### 2.2. Preparation of Calcitriol Stock Solution

**Figure 2:**
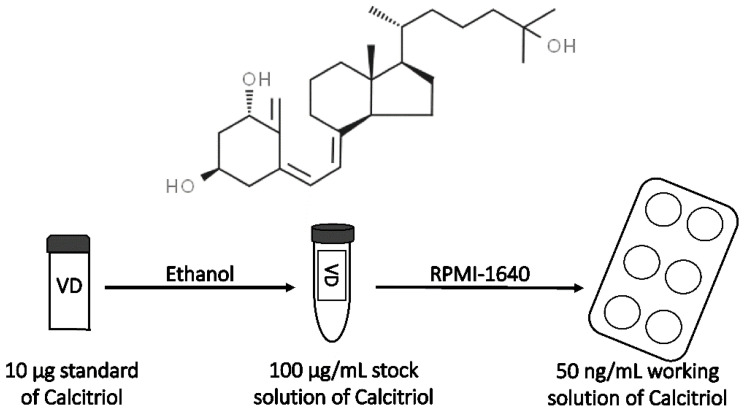
Preparation of calcitriol working solution and its chemical structure.

Lyophilized VD3 (Figure 2) standard (10 µg) was dissolved in EtOH in order to prepare a stock solution. The final concentration of EtOH in the medium for cell culture was 0.0025%. The VD3 stock solution was aliquoted and stored at −20 °C until use.

### 2.3. Preparation of Fatty Acid Solution and Its Control

The FFA stock solution was prepared in EtOH by using oleic and palmitic acid in a 2:1 ratio with a final concentration of 0.2 M. The mix of FFA and their ratio were chosen based on previous experiments, where the cytotoxicity of palmitic acid (saturated fatty acid) is blunted in the mixture with oleic acid (mono-unsaturated fatty acid) in order to reproduce a more in vivo situation as oleic and palmitic acids are abundant in human plasma [47,48]. The solution was stored in a dark tube at −20 °C. On the day of use, FFA stock solution was added to a Hanks solution with 10% BSA in order to obtain a water-soluble solution (FFA/BSA solution) with an FFA final concentration of 5 mM. The latter solution was prepared through an incubation period of 30 min at 37 °C with occasional shaking. The final concentration of FFA in the cell culture medium was 500 μM, while the negative control cell culture medium contained an equal volume of EtOH instead of the FFA solution. The final EtOH and BSA concentrations in the cell media were 0.1% each.

### 2.4. THP-1 Cell Culture

The THP-1 cell line (human monocytic leukemia) was obtained from the American Type Culture Collection (Manassas, VA, USA). The cell culture medium was RPMI-1640 supplemented with 10% of heat-inactivated FBS, 1% sodium pyruvate, 1% HEPES, and 0.1% gentamicin. Every third day during cell growth, the THP-1 cells were subcultured by withdrawing a volume of medium containing cells from the culture flask and adding complete fresh medium to obtain the appropriate seeding density of 3 × 10^5^ cells/mL. The THP-1 cell line maintains monocytic characteristics for over 14 months of continuous growth [49]. In our study, a maximum period of 2 months was adopted for cell growth, which corresponds to a passage number between 5 and 15.

### 2.5. Viability Assay

The Trypan Blue dye exclusion assay was used to assess the toxicity of the compounds tested on THP-1-derived macrophages. The cell count was performed by using a TC20TM automated cell counter and dual-chamber cell counting slides (BIORAD, Segrate, Milan, Italy). The cells were resuspended in fresh complete medium in order to reach 4 × 10^5^ cells/mL as the final concentration, and 1 mL of cell suspension (4 × 10^5^ cells) was added into each well of a 12-well plate. Monocytes were differentiated into macrophages and incubated with VD3 at different concentrations (from 0.01 to 100 μM) in the presence of FFA/BSA solution (500 μM) for 24 h. Afterward, macrophages were trypsinized and resuspended in their supernatants (in order to also include dead cells), and viability was analyzed by the Trypan Blue dye exclusion assay. Each compound and concentration was assessed in triplicate over three independent experiments (Table 1). Triton X-100 (0.1%) was used as positive control. The absolute viability is expressed as the percentage of viable cells out of all counted cells.

### 2.6. Lipid Accumulation Assay in Macrophages

THP-1 cells were maintained in complete RPMI-1640 medium at 37 °C and 5% CO_2_. Once a proper cell number was reached, the differentiation into macrophages was induced by exposure to 5 ng/mL PMA for 72 h. Undifferentiated cells were removed by washing with Hanks’ solution since THP-1-derived macrophages became adherent to the culture flask surface. Afterward, in order to collect the differentiated macrophages, cells were trypsinized by incubating with 3 mL trypsin (0.05%)–EDTA (0.53 mM) at 37 °C and 5% CO_2_ for 2 min. The addition of 2 mL of complete RPMI-1640 medium allowed us to inactivate the trypsin. Falcon tubes were used to collect and centrifuge the cells (mod. Eppendorf 5804R Centrifuge, Milan, Italy) at 250× *g* for 5 min. Then, the cells were quantified by using a TC20TM automated cell counter and resuspended in fresh complete medium in order to reach 2.5 × 10^5^ cells/mL as the final concentration. The adhesion of macrophages was carried out by using a black 96-well plate and by adding 200 µL of cell suspension (equivalent to 5 × 10^4^ cells) into each well. Cells were incubated at 37 °C and 5% CO_2_ for 24 h. Then, the medium was replaced with new RPMI-1640 containing 500 μM of FFA and VD3 at different concentrations, and macrophages were incubated for 24 h at 37 °C and 5% CO_2_. The concentrations of VD3 were 0.01, 0.1, 10, 50 and 100 µM. The Nile Red dye was used to measure the intracellular lipid accumulation [47], which is extensively fluorescent in lipid-rich environments. In detail, macrophages were washed with Hanks’ solution and then stained using 0.5 μg/mL of Nile Red dissolved in Hanks’ solution supplemented with 0.01% Pluronic F127 and 0.01% Pluronic F127 and maintained in incubation for 15 min at 37 °C and 5% CO_2_. Cells were washed twice with Hanks’ solution (200 μL) and soaked in 100 μL of new Hanks’ solution prior to the fluorescence analysis. For the quantification, a fluorescence spectrophotometer (mod. F200 Infinite, TECAN, Milan, Italy) was used and the fluorescence determined (excitation: 544 nm, emission: 590 nm). Each concentration of VD3 was derived from 3 independent experiments in which each concentration was tested 7 times.

### 2.7. Protein Quantification by Enzyme-Linked Immunosorbent Assay (ELISA)

In order to collect a sufficient number of cells, THP-1-derived macrophages were seeded in 6-well plates (1 × 10^6^ cells/well) instead of 96-well plates (5 × 10^4^ cells/well). In conformity with the manufacturer’s protocol, the supernatant of cell culture was collected for PPAR-γ1, CD36, and ABCA1 analyses; cell extract was used for C/EBPβ and CPT-1A analysis. We used cell culture supernatants for PPAR-γ1, CD36, and ABCA1 measurements because it was within the manufacturer’s specification of the assays and because soluble CD36 and ABCA1 in plasma are used as biomarkers of cardiovascular diseases in humans [50,51]. The supernatant was centrifuged at 500× *g* for 10 min at 4 °C. Protein extraction was performed by placing cells on ice, washing with cold PBS, and incubating for 30 min on ice with an extraction buffer. The cell lysate was centrifuged at 4500× *g* for 20 min at 4 °C. Aliquots of supernatants and cell lysate were stored at −80 °C until analysis. ELISA kits were used to assess the protein levels of C/EBPβ, PPAR-γ1, CD36, CPT-1A, and ABCA1 (Cat No. were MBS2709324, MBS263089, MBS2020315, MBS166979, and MBS267210, respectively; MyBioSource, Inc. San Diego, CA, USA). The analyses were conducted in triplicate, and the results were derived from three independent experiments.

### 2.8. Data Analysis

The effect of VD3 supplementation on cell viability, the lipid accumulation process, and the protein levels of C/EBPβ, PPAR-γ1, CD36, CPT-1A, and ABCA1 was evaluated by one-way ANOVA using STATISTICA software (Statsoft Inc., Tulsa, OK, USA). Differences between treatments were identified by the least significant difference (LSD) test by setting the level of statistical significance at *p* < 0.01. Results are reported as means ± standard deviation (SD).

## 3. Results

### 3.1. Effect of VD3 on Cell Viability

Table 1 presents the effect of the different VD3 concentrations (0.1–100 nM) tested for 24 h in the presence of FFA/BSA solution (500 μM) on the cellular viability measured by Trypan Blue exclusion assay. The control condition is represented by cells in their normal growth medium without VD3 and FFA. VD3 + FFA did not reduce the cell viability, which remained higher than 90% (*p* > 0.05), while, as expected, the addition of Triton X-100 (0.1%) induced a significant reduction (−77.9%, *p* < 0.0001).

### 3.2. Effect of VD3 on Lipid Accumulation in THP-1 Derived Macrophages

**Figure 3:**
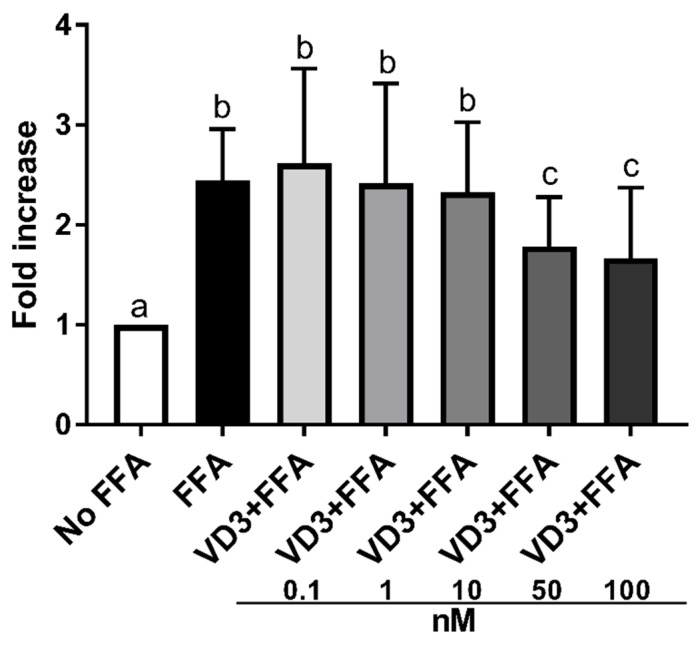
Effect of different concentrations (0.1–100 nM) of VD3 on cell lipid accumulation. Data are reported as a fold increase and mean ± standard deviation. ^a,b,c^ Bar graphs with different letters are significantly different (*p* ≤ 0.01). No FFA: no free fatty acids (control); FFA: free fatty acids (500 μM); VD3: calcitriol + free fatty acids (500 μM).

Figure 3 depicts the effect of the supplementation with VD3 on lipid accumulation in THP-1-derived macrophages. Exposure to 500 μM of FFA significantly increased (*p* < 0.01) the lipid accumulation in macrophages compared to cells without treatment (No FFA). Treatment with 50 and 100 nM of VD3 significantly lowered (*p* < 0.01) lipid accumulation in macrophages compared to the positive control (FFA exposure only). In particular, the size of the effect was similar, −27% and −32%, respectively, for VD3 at 50 and 100 nM. Since the effect on lipid accumulation was comparable at 50 and 100 nM, the experiments on gene expression were performed by using the lowest concentration.

### 3.3. Effect of VD3 on PPAR-γ1 Protein Levels

**Figure 4:**
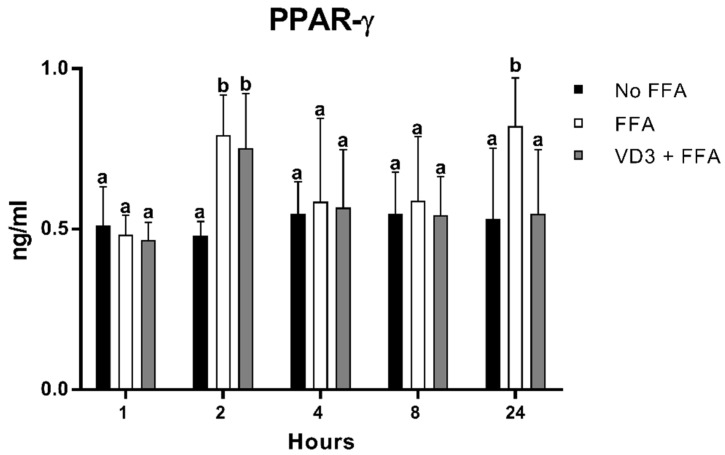
Effect of VD3 supplementation on PPAR-γ1 protein levels. Data are reported as mean  ± standard deviation. ^a,b^ Bar graphs with different letters are significantly different (*p* ≤ 0.01). No FFA: no free fatty acids (control); FFA: free fatty acids (500 µM); VD3: calcitriol (50 nM).

The results of the protein expression kinetic of PPAR-γ1 after the administration of VD3 at a concentration of 50 nM are depicted in Figure 4. A statistically significant increase (*p* < 0.01) in PPAR-γ1 protein levels was documented at 2 and 24 h following the incubation with FFA compared to No FFA (+65.3% and +54.4%, respectively). The treatment with VD3 (VD3 + FFA) induced an increase in PPAR-γ1 levels at 2 h compared to the negative control (+57%; *p* < 0.01), while the exposure for 24 h determined a reduction in PPAR-γ1 protein level (−33.4%; *p* < 0.01). No difference was found when considering the negative control (No FFA) or the other time points analyzed.

### 3.4. Effect of VD3 on CD-36 Protein Levels

**Figure 5:**
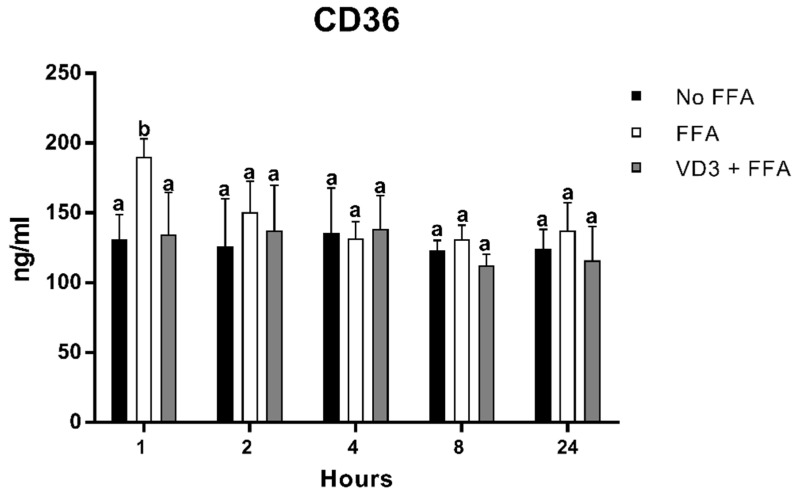
Effect of VD3 supplementation on CD36 protein levels. Data are reported as mean  ±   standard deviation. ^a,b^ Bar graphs with different letters are significantly different (*p* ≤ 0.01). No FFA: no free fatty acids (control); FFA: free fatty acids (500 µM); VD3: calcitriol (50 nM).

Figure 5 depicts the result of FFA and VD3 treatments on the expression of CD-36 in macrophages at different time points. There was a statistically significant increase of CD-36 protein expression after 1 h of FFA incubation compared to the negative control (+45.2%; *p* < 0.01), while, at the same time point, the treatment with VD3 in the presence of FFA was able to counteract the increase of CD-36 protein levels induced by FFA administration (−29.1%; *p* < 0.01). No statistically significant difference was observed between the negative control and the VD3 + FFA. No difference was documented for the other time points analyzed.

### 3.5. Effect of VD3 on C/EBPβ Protein Levels

**Figure 6:**
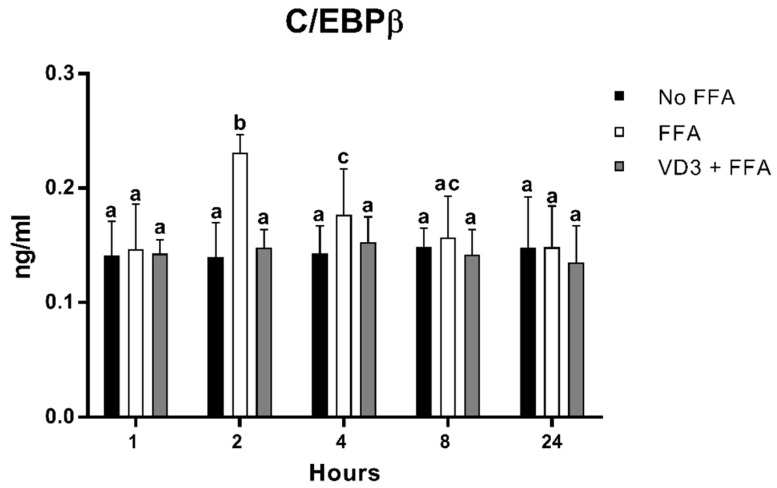
Effect of VD3 supplementation on C/EBPβ cell protein levels. Data are reported as mean ±  standard deviation. ^a,b,c^ Bar graphs with different letters are significantly different (*p* ≤ 0.01). No FFA: no free fatty acids (control); FFA: free fatty acids (500 µM); VD3: calcitriol (50 nM).

Figure 6 shows the protein levels of C/EBPβ at different time points after the treatment with VD3 (50 nM) and FFA (500 µM). A statistically significant increase in C/EBPβ protein levels was documented following cell incubation with FFA (positive control) compared to negative control (No FFA) at 2 h (+65.2%; *p* < 0.01) and 4 h (+23.9%; *p* < 0.01). The treatment with VD3 inhibited the FFA-induced increase in C/EBPβ protein level at both 2 h (−35.9%; *p* < 0.01) and 4 h (−16.1%; *p* < 0.01) compared to only FFA. No difference was documented with respect to No FFA.

### 3.6. Effect of VD3 on CPT-1A Protein Levels

**Figure 7:**
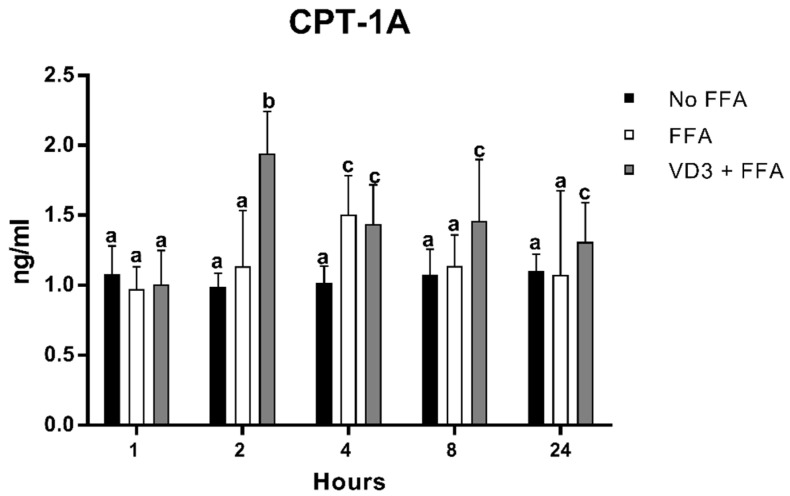
Effect of VD3 supplementation on PPAR-γ1 protein levels. Data are reported as mean ± standard deviation. ^a,b,c^ Bar graphs with different letters are significantly different (*p* ≤ 0.01). No FFA: no free fatty acids (control); FFA: free fatty acids (500 µM); VD3: calcitriol (50 nM).

The results of FFA and VD3 administration on CPT-1A protein expression kinetics are shown in Figure 7. The earliest statistically significant modification occurred after 2 h of treatment with VD3, which was an increase of CPT-1A protein levels compared to both negative control and the FFA condition (+96.7% and +71.4%, respectively; *p* < 0.01). No significant difference was documented between the negative control and the positive control at the same time point. After 4 h, the incubation with FFA increased the CPT-1A protein levels, similar to the condition of VD3 + FFA (not statistically different) but significantly higher than the negative control (+48%; *p* < 0.01). However, at 8 and 24 h, the protein levels of CPT-1A after FFA treatment returned to baseline. The addition of VD3 (VD3 + FFA) was able to maintain significantly higher levels of CPT-1A at 8 and 24 h compared to the negative control (+35.5% and + 18.8%, respectively; *p* < 0.01).

### 3.7. Effect of VD3 on ABCA1 Protein Levels

**Figure 8:**
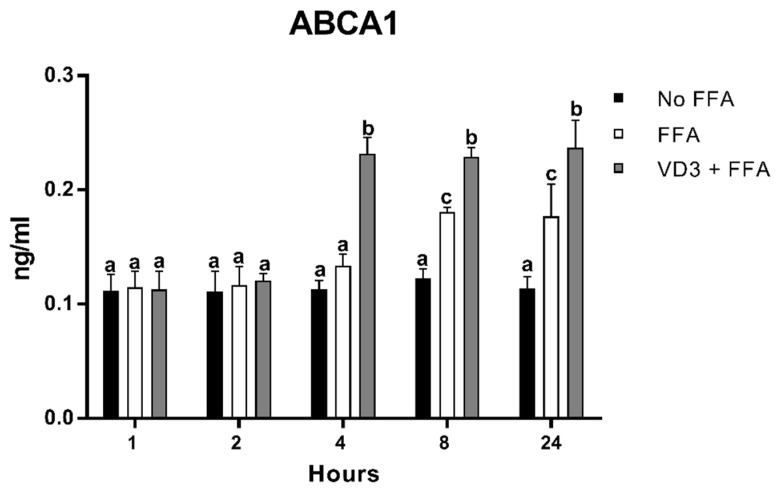
Effect of VD3 supplementation on ABCA1 protein levels. Data are reported as mean ± standard deviation. ^a,b,c^ Bar graphs with different letters are significantly different (*p* ≤ 0.01). No FFA: no free fatty acids (control); FFA: free fatty acids (500 µM); VD3: calcitriol (50 nM).

The results of the protein expression kinetic of ABCA1 after VD3 and FFA treatments are shown in Figure 8. THP-1-derived macrophages incubated with FFA + VD3 had significantly higher protein levels of ABCA1 compared to the positive control (only FFA) at 4 h (+72.5%; *p* < 0.01), 8 h (+26.8%; *p* < 0.01), and 24 h (+33.6%; *p* < 0.01). The treatment with FFA alone was able to increase ABCA1 in a significant way compared to the negative control only at 8 h (+46.5%; *p* < 0.01) and 24 h (+54.7%; *p* < 0.01), although to a lower extent compared to the addition of VD3.

## 4. Discussion

In the present study, we document the capacity of VD3 to mitigate the accumulation of lipids in THP-1-derived macrophages exposed to FFA. This effect seems to be attributed to an upregulation of several proteins involved in the metabolism of fatty acids, such as C/EBPβ, PPAR-γ1, CD36, CPT-1A, and ABCA1, supporting the potential anti-atherogenic activity of VD3.

The role of VD in the modulation of lipid metabolism has been evaluated in different studies [52,53,54,55,56]. The results seem to support the contribution of VD in counteracting lipid accumulation in THP-1-derived macrophages and other cell lines in line with our findings. For example, Riek and colleagues [52] showed that VD3 supplementation (100 nM for 5 days) prevented foam cell formation through the reduction of cholesterol deposition and the enhancement of cholesterol efflux in human monocyte-derived macrophages from adults with type 2 diabetes mellitus (T2DM). Moreover, the same authors [53] documented that the deletion of the vitamin D receptor (VDR) in mice accelerated the atherosclerotic process, probably due to an increase of lipid-laden macrophages in atheroma as well as higher cholesterol uptake and deposition in foam cells. Yin and coworkers [54] observed lower levels of lipid accumulation in THP-1-derived macrophages incubated with oxidized LDL (ox-LDL; 50 μg/mL for 48 h) and VD3 (10 nM for 24 h) compared to the same model, but without VD3 and only treated with ox-LDL. In addition, the use of VDR antagonists determined the inhibition of cholesterol efflux, supporting the anti-atherogenic role of VD3 through the interaction with its ubiquitous receptor. The reduction in lipid droplet accumulation has also been shown in a murine model of 3T3-L1 preadipocytes [55]. Li et al. demonstrated that VD3 treatment was able to counteract the storage of lipids by 79% compared to the control in 3T3-L1 cells, although they used higher concentrations (1 µM) and times of exposure (up to 60 h) compared to our experimental conditions. Finally, Chang and colleagues [56] documented a reduction in lipid accumulation and an increase in lipolysis after treating 3T3-L1 adipocytes with 100 nM VD3 for 24 h.

The role of VD3 in the modulation of atherosclerosis is not well understood. The main mechanisms potentially involved in this effect include the up- or downregulation of the expression of genes embroiled in the lipid uptake and efflux. Among transcriptional pathways, PPAR-γ1 has been recognized to play a pivotal role in the control of FA metabolism. FA and FA-derived compounds are strong ligands for PPAR γ1 [57]. In fact, in our experimental conditions, we found that macrophages incubated with FFA increased PPAR-γ1 levels after 2 and 24 h. The addition of VD3 was able to inhibit PPAR-γ1, but this effect was only at 24 h. This lack of PPAR-γ1 overexpression could be due to a resolution of intracellular lipid accumulation, in line with what was previously published [58]. The effect of VD3 on PPAR-γ1 and lipid accumulation has been investigated in different in vitro models, with results in line with our findings [55,59,60,61]. For example, Oh and colleagues [60] demonstrated the capacity of VD3 (100 nM) to inhibit the expression of PPAR-γ1 in macrophages and to reduce cholesterol uptake and foam cell formation. Scrimieri and coworkers [59] showed that VD3 (20 nM) downregulated PPAR-γ1 and decreased intracellular fat storage accumulation, induced by 30 mM D-glucose for 24 h, in endothelial cells. Likewise, Li et al. [55] demonstrated the prevention of lipid droplet accumulation in mouse 3T3-L1 preadipocytes following VD3 treatment (1 µM, 60 h), attributing the anti-adipogenic effect to the capacity to decrease the expression of PPAR-γ1.

An overexpression of PPAR-γ1 leads to an upregulation of CD-36, a protein transporter recognized to promote the cellular uptake of ox-LDL and FFA. We have found that FFA increased CD36 expression at 1 h from their administration; however, this effect was only temporary, suggesting that it was strictly dependent on FFA availability. The addition of VD3 was able to inhibit CD36 overexpression, which results in major control of FFA uptake into the cell. Similar findings were also reported by other authors. For example, Szeto and colleagues [62] demonstrated that LDLR^−/−^VDR^−/−^ mice supplemented with a high fat–high cholesterol diet for 8–12 weeks experienced higher lipid accumulation in macrophages due to an overexpression of CD36, compared with LDLR^−/−^ mice. Similarly, Oh et al. [53] found that the bone marrow transplant of VDR into KODMAC mice (in which VDR was inactivated in myeloid cells) decreased foam cell formation and suppressed atherosclerosis. In addition, the authors observed that macrophages treated with 100 nM of VD3 expressed lower levels of CD36 and reduced cholesterol uptake. In a previous study [60], the same authors documented that VD3 supplementation downregulated CD36 by preventing cholesterol uptake and counteracting foam cell formation in isolated macrophages from diabetic patients. Conversely, Alizadeh et al. [63] observed higher levels of CD36 expression in the aorta of adult male VD/diabetic rats after 4-week supplementation with higher doses of VD3 (5000 IU/kg).

The intracellular concentration of FAs brings an overexpression of not only PPAR-γ but also of other genes involved in the lipid metabolism, including those related to the inflammatory response, such as C/EBPβ, and the beta-oxidation, such as CPT-1A, as documented by their temporary upregulation (at 2 and 4 h for C/EBPβ and at 4 h for CPT-1A). The incubation of VD3 with FFA blunted the overexpression of C/EBPβ, while it maintained upregulated CPT-1A from 2 h up to 24 h compared to control, suggesting that VD3 stimulates the lipid metabolism by reducing intracellular lipid accumulation and increasing the oxidation process; meanwhile, VD3 was also able to blunt the inflammatory response at the levels of macrophages. To the best of our knowledge, the role of VD3 on C/EBPβ and CPT-1A has been poorly investigated, in particular at the levels of macrophages, but the results seem to be in line with our observations [64,65,66,67]. Specifically, Blumberg et al. [64] reported that the treatment with VD3 (1–100 nM) in the preadipocytes 3T3-L1 cell line reduced the expression of C/EBPβ and upregulated the C/EBPβ corepressor, determining the blocks of adipogenesis and the reduction of lipid accumulation. Chang and colleagues [67] showed significantly higher levels of CPT-1 in muscle cells treated with VD3 (100 nM; 24 h). Scrimieri and coworkers [59] observed an upregulation of CPT-1A induced by VD3 (20 nM) in endothelial cells incubated with a high-glucose concentration (30 mM d-glucose for 24 h). The effect of VD3 on CPT-1A has also been investigated in animal models, showing similar findings following the co-administration of a high-fat/high-sugar diet and VD3 [68,69].

The cellular uptake of FFA and their accumulation, together with the upregulation of PPAR-γ, is reported to induce the transcription of different cell membrane proteins involved in the lipid efflux, such as ATP-binding cassette transporter ABCA1 [45]. Here, we documented that FFA increased in ABCA1 protein levels at 8 and 24 h; at the same time points, the supplementation with VD3 augmented the ABCA1 protein levels, also stimulating an early expression at 4 h compared to the positive control (only FFA administration). This increase in ABCA1 protein levels could represent another anti-atherogenic potential mechanism of VD3, consisting of a major cellular lipid efflux and a lower intracellular lipid level. In addition, several studies have reported that ABCA1 upregulation is recognized to have an anti-inflammatory function in a diverse range of diseases where inflammation is an underlying pathogenic mechanism [70,71]. These results, together with those reported for C/EBPβ, seem to also support the potential anti-inflammatory activity of VD3. Coherently with our findings, Yin and colleagues [38] found that VD3 (10 nM) led to an upregulation, at 12 and 24 h, of ABCA1 expression in THP-1-derived macrophages that had been stimulated with ox-LDL (50 μg/mL, 48 h) prior to the VD3 supplementation. This delay in ABCA1 activation between our and their study could be attributed to a difference in VD3 concentrations. The same authors found that Yucatan microswine fed for 48 weeks with a high cholesterol vitamin D-deficient (0 IU/d) diet had decreased ABCA1 liver levels, while the supplementation with vitamin D (3000 IU/d) increased HDL plasma levels and reduced liver cholesterol accumulation. Another recent study documented that the supplementation with VD3 (1 µM; at 6 and 24 h) stimulated ABCA1 expression in murine dermal fibroblasts and immortalized human epidermal keratinocytes (HaCaT cells) [72].

The present study has certain limitations. First, the statistical interaction between VD3 and FFA has not been assessed, which would have been possible by including a group with only VD3-treated cells. This does not allow us to verify whether VD3 alone is able to upregulate these genes in normal conditions (without stimulation) or if the effects are attributable exclusively to a stimulus (e.g., when administering FFA). Second, we made the decision to analyze only selected proteins involved in lipid metabolism instead of providing an overview of all genes potentially involved in such modulation. Third, the expression of proteins was assessed in cell extracts or cell culture supernatants. This was done according to the specification of the assay kits. However, a more elaborate analysis could include measurements of mRNA levels of the corresponding proteins or even protein levels by Western blot or immunocytochemistry. This would provide information regarding the mRNA-protein correspondence. Fourth, there was a lack of quantification of the specific lipids entered, accumulated, and leaked from the cells, which could sustain the upregulation observed. Finally, there was an absence of markers related to inflammation (e.g., interleukins, TNF-α, INF-γ) or oxidative stress (e.g., ox-LDL, malondialdehyde) that would be able to substantiate our findings.

## 5. Conclusions

In conclusion, based on our results, VD3 has a beneficial effect on lipid metabolism by affecting the production of different proteins involved in the cellular uptake, transport, oxidation, and efflux of lipids in macrophages. Thus, the maintenance of an optimal vitamin D nutritional status could represent a relevant strategy to reduce the risk of CVDs. PPAR-γ1 seems to represent a key regulator in the modulation of lipid metabolism due to its direct interaction with the other proteins involved, such as C/EBPβ, CD36, CPT-1A, and ABCA1. However, since we cannot exclude that VD3 may also influence lipid metabolism through the up/downregulation of other potential target genes, further mechanistic studies are encouraged to corroborate the actual findings and explore new potential metabolic pathways, including those related to oxidative stress and inflammation, in order to better elucidate the biological role of VD3. In this regard, the combination with an integrated multi-omic approach (e.g., lipidomic, metabolomic, and transcriptomic) would allow better comprehension of the signaling pathways involved in this complex landscape.

## Figures and Tables

**Table 1:** Cell Viability.

Condition	VD3 Concentration (nM)	% of Cell Viability *
Control	-	90.7 ± 1.5
Triton X-100	-	20 ± 6.2 **
FFA	-	92.1 ± 2.2
FFA + VD3	0.1	91.3 ± 2.6
FFA + VD3	1	90.9 ± 1.8
FFA + VD3	10	93.4 ± 3.3
FFA + VD3	50	92.5 ± 2.4
FFA + VD3	100	90.3 ± 1.3

Legend: Results derived from three independent experiments, in which each concentration was tested in triplicate. Data are reported as the mean ± standard deviation. * The total number of cells was not different between the exposure groups (i.e., 4 × 10^5^ cells/well, corresponding to a plating efficiency higher than 90%). ** *p* ≤ 0.01 compared with control. VD3: calcitriol; FFA: free fatty acids.
